# Expression Profiling of Calcium Channels and Calcium-Activated Potassium Channels in Colorectal Cancer

**DOI:** 10.3390/cancers11040561

**Published:** 2019-04-19

**Authors:** Sajida Ibrahim, Hassan Dakik, Christophe Vandier, Romain Chautard, Gilles Paintaud, Frédéric Mazurier, Thierry Lecomte, Maxime Guéguinou, William Raoul

**Affiliations:** 1 Université de Tours, EA 7501 GICC, 37032 Tours CEDEX 01, France; sajida.ibrahim@etu.univ-tours.fr (S.I.); hassan.dakik@univ-tours.fr (H.D.); romain.chautard@etu.univ-tours.fr (R.C.); gilles.paintaud@univ-tours.fr (G.P.); thierry.lecomte@univ-tours.fr (T.L.); maxime.gueguinou@univ-tours.fr (M.G.); 2 CNRS ERL 7001 LNOx, 37032 Tours CEDEX 01, France; 3 Inserm UMR 1069, Nutrition Croissance et Cancer (N2C), 37032 Tours CEDEX 01, France; christophe.vandier@univ-tours.fr; 4 CHRU de Tours, Department of Hepato-Gastroenterology and Digestive Oncology, 37044 Tours CEDEX 09, France; 5 CHRU de Tours, Department of Medical Pharmacology, 37044 Tours CEDEX 9, France

**Keywords:** colorectal cancer, calcium channels, calcium-activated potassium channels, calcium signaling, prognosis, survival

## Abstract

*Background*: Colorectal cancer (CRC) is a highly devastating cancer. Ca^2+^-dependent channels are now considered key regulators of tumor progression. In this study, we aimed to investigate the association of non-voltage gated Ca^2+^ channels and Ca^2+^-dependent potassium channels (KCa) with CRC using the transcriptional profile of their genes. *Methods*: We selected a total of 35 genes covering KCa channels *KCNN1*–*4*, *KCNMA1* and their subunits *KCNMB1*–*4*, endoplasmic reticulum (ER) calcium sensors *STIM1* and *STIM2*, Ca^2+^ channels *ORAI1–3* and the family of cation channels TRP (*TRPC1–7*, *TRPA1*, *TRPV1/2,4–6* and *TRPM1–8*). We analyzed their expression in two public CRC datasets from The Cancer Genome Atlas (TCGA) and GSE39582. *Results*: *KCNN4* and *TRPM2* were induced while *KCNMA1* and *TRPM6* were downregulated in tumor tissues comparing to normal tissues. In proximal tumors, *STIM2* and *KCNN2* were upregulated while *ORAI2* and *TRPM6* were downregulated. *ORAI1* decreased in lymph node metastatic tumors. *TRPC1* and *ORAI3* predicted poor prognosis in CRC patients. Moreover, we found that *ORAI3/ORAI1* ratio is increased in CRC progression and predicted poor prognosis. *Conclusions*: KCa and Ca^2+^ channels could be important contributors to CRC initiation and progression. Our results provide new insights on KCa and Ca^2+^ channels remodeling in CRC.

## 1. Introduction

Colorectal cancer (CRC) is the third most common cancer in western countries, with a highly metastatic proportion at diagnosis (20% to 25%) [1,2]. Understanding the mechanisms behind tumor migration, invasion and metastasis is essential to the search for drugs that will help prevent the spread of the disease and thereby reduce related mortality. Indeed, cell migration and invasion are Ca^2+^-dependent processes orchestrated through many Ca^2+^-sensitive effector molecules in both normal and pathological conditions. However, even though the role of Ca^2+^ signaling has long been suspected in these processes, ion channel proteins were largely understudied until recently [3,4]. Over the last two decades, it became evident that Ca^2+^ channels act as novel and important regulators of specific steps in tumor progression covering all major hallmarks of cancer progression [5,6]. Non-voltage gated Ca^2+^ channels are the major Ca^2+^ entry pathways in non-excitable cells and can be divided into two groups: Store Operated Channels (SOCs) and Store-independent Ca^2+^ channels (SICs).

The concept of Store Operated Calcium Entry (SOCE) was proposed by Putney three decades ago [7]. Briefly, upon stimulation, phospholipase C (PLC)-linked receptors produce inositol trisphosphate (IP3), which then interacts with IP3 receptors (IP3R) on the membrane of the endoplasmic reticulum (ER) and induces the release of Ca^2+^ [8]. The decrease in ER Ca^2+^ concentration is followed by the activation of two transmembrane proteins located in the ER membrane: stromal interaction molecule 1 (STIM1) and STIM2. The dissociation of Ca^2+^ from the EF hand domains of STIM1 and STIM2 results in conformational changes that enable them to activate ORAI and TRPC1 channels in the plasma membrane [9].

On the other hand, SICs include three different subtypes of channels: Receptor Operated Channels (ROCs), Second Messenger Operated Channels (SMOCs) and Constitutive Ca^2+^ Channels (CCCs). While CCCs are active without any stimulation [10], ROCs and SMOCs are active upon stimulation. ROCs, i.e., the P2X receptor, are directly activated in response to extracellular signals. SMOCs, i.e., TRP, are activated by intracellular ligands, such as diacyglycerol (DAG) or phospholipase A2 (PLA2).

Moreover, Shuttleworth and colleagues have identified a Ca^2+^-conductance activated by low concentration of exogenous AA and named it “Arachidonate Regulated Ca^2+^ Channel” (ARC channel) [11]. ARC channels were also found to be activated by low concentrations of agonists in which oscillatory Ca^2+^ signals are induced in murine parotid and pancreatic acinar cells [12]. It was later suggested that the ARC channel is formed by a combination of ORAI1 and ORAI3 subunits and regulated by STIM1 that constitutively resides in the plasma membrane [13,14].

The increase of cytosolic Ca^2+^ concentration through SOCs and SICs channels activates Ca^2+^-activated potassium channels, KCa, that are ranked according to their conductance: small-conductance potassium channels (SKCa) including KCa2.1 (SK1), 2.2 (SK2) and 2.3 (SK3) coded by *KCNN1*, *KCNN2* and *KCNN3* genes, respectively; intermediate-conductance potassium channels (IKCa), including KCa3.1 (SK4) coded by the *KCNN4* gene; and big-conductance potassium channels, including BKCa coded by the *KCNMA1* gene [15]. Indeed, the activation of KCa channels in non-excitable cells increases Ca^2+^ entry through SOCs and SICs by increasing the Ca^2+^ driving force. This positive feedback loop induced by KCa contributes to an increase in cytosolic Ca^2+^ concentration and amplifies the effects of Ca^2+^ signaling. However, the normal functions of the KCa channels are hijacked by cancer cells in order to promote their proliferation and metastasis. It became evident that an association of KCa and Ca^2+^ channels is found in cholesterol enriched nanodomains (also named lipid rafts) in cancer cells and contributes to cancer-associated functions, such as cell proliferation, migration and metastasis capacity [16]. In normal colonic cells, SOC is mediated by STIM1, STIM2 and ORAI1 [17], while in CRC cells, SOCE is mediated by STIM1, ORAI1, TRPC1 and SK3 [18]. Noteworthy, targeting the SK3/TRPC1/ORAI1 complex inhibited CRC cell migration [19,20]. Moreover, Villalobos and colleagues showed that the remodeling of SOCE is associated with increased expression of ORAI1, STIM1 and TRPC1 in HT29 CRC cells compared to immortalized NCM460 normal colon epithelial cell line [17]. Therefore, Villalobos et al. proposed that SOCE could be a novel key player in CRC and its inhibition by salicylate and that other nonsteroidal anti-inflammatory drugs (NSAIDs) could be a possible mechanism of cancer chemoprevention [21]. Recently, Moccia and colleagues showed that STIM1 and ORAI3 are upregulated in metastatic CRC cells with reduced constitutive Ca^2+^ entry and SOCE compared to primary CRC cells [22].

In this study, we aimed to investigate the transcriptional profile of genes coding for non-voltage gated Ca^2+^ channels and Ca^2+^-dependent potassium channels in CRC. For this purpose, we selected a total of 35 genes covering KCa channels *KCNN1–4*, *KCNMA1* and its subunits *KCNMB1–4*, as well as ER Ca^2+^ sensors STIM1 and STIM2, Ca^2+^ channels ORAI1–3 and the family of cation channels TRP (*TRPC1–7*, *TRPA1*, *TRPV1/2,4–6* and *TRPM1–8*) (Table S1). We analyzed their expression in two well-annotated public CRC datasets from The Cancer Genome Atlas (TCGA, RNAseq dataset) and *La Ligue Contre le Cancer* (GSE39582, microarray dataset). First, we studied the association of gene expression profiles with CRC initiation by comparing the expression between normal and tumor tissues. Next, we investigated their co-expression and their association with tumor primary sites, lymph node metastases and prognostic capacity. We found that KCa and Ca^2+^ channels could be important contributors to CRC initiation and progression. Our results also provide new insights about Ca^2+^ remodeling in CRC.

## 2. Results

### 2.1. Gene Expression of KCa and Ca^2+^ Channels in Normal Mucosa versus CRC Tissues

**Figure 1:**
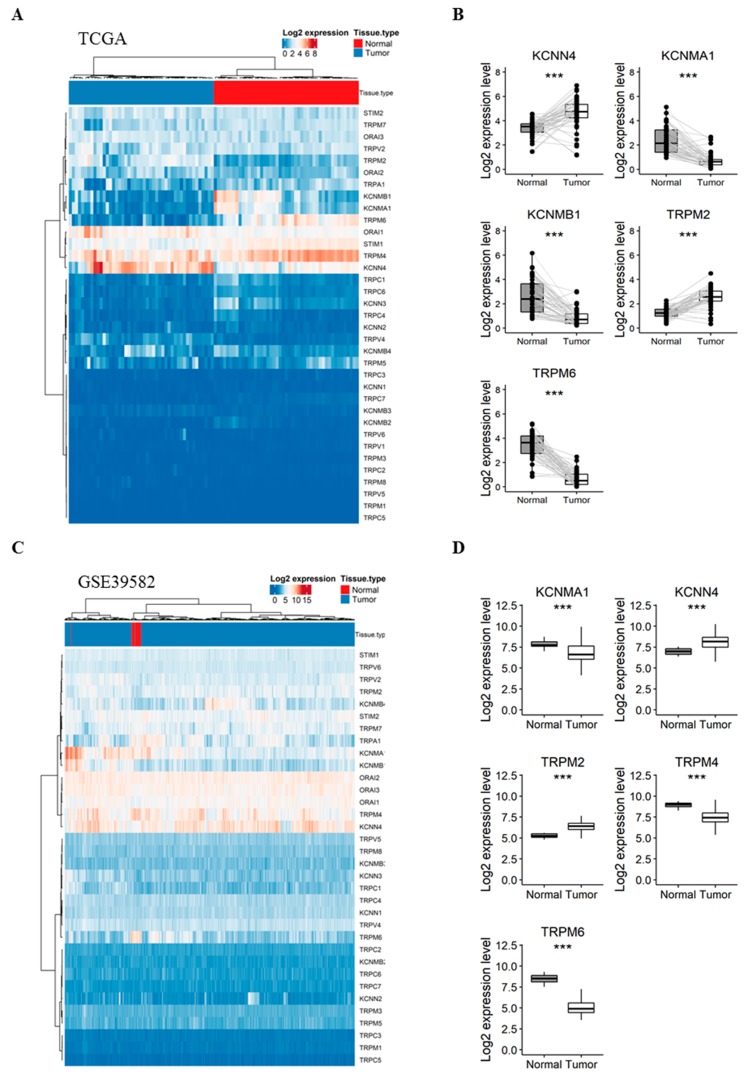
Comparison of the gene expression between CRC tumors vs. adjacent normal mucosa in TCGA (**A**,**B**) and GSE39582 (**C**,**D**) patients. (**A**) Heatmap presenting the expression profile of 35 Ca^2+^ and KCa channels coding-genes between CRC and matched normal tissues from the TCGA dataset; (**B**) Boxplots of differentially expressed genes between normal and CRC tissues in the TCGA dataset; (**C**) Heatmap presenting the expression profile of 34 Ca and KCa channels coding-genes between CRC and unmatched normal tissues from GSE39582 dataset; (**D**) Boxplots of differentially expressed genes between normal and CRC tissues in the GSE39582 dataset. Hierarchical clustering was done using Euclidean distance and average linkage method from both genes and samples. Signed-rank Wilcoxon’s test and unpaired t-test were used to perform pairwise comparisons between CRC and normal tissues in the TCGA and GSE39582 datasets, respectively and were followed by BH adjustment, respectively. (***: BH-adjusted *p* < 0.001). CRC: colorectal cancer; TCGA: The Cancer Genome Atlas; Ca: calcium; KCa: Ca^2+^-dependent potassium channels. BH: Benjamini-Hochberg.

Our first aim was to analyze whether the expression of selected genes varies between normal colorectal mucosa and CRC tissues. Therefore, we compared their expression in 47 pairs of matched normal mucosa and tumor tissues from the TCGA dataset. Interestingly, the expression profile of the 35 genes was sufficient to perfectly discriminate normal samples from tumor ones when performing unsupervised hierarchical clustering (Figure 1A). Interestingly, five genes were highly deregulated between the two tissues (Figure 1B): (i) *KCNN4* and *TRPM2* were upregulated in tumor tissues with a fold-change of 1.24 and 1.28 (Table S2), respectively; (ii) while *KCNMA1*, *KCNMB1* and *TRPM6* were significantly downregulated in tumor tissues by −1.66, −1.73 and −2.76 fold, respectively (Table S2).

To validate these results, we next compared the expression profile of the genes in unmatched normal (*n* = 19) and tumor (*n* = 566) tissues from the GSE39582 dataset. The studied genes were again efficient to separate normal and tumor samples into distinct clusters (Figure 1C). Pairwise analysis showed that five genes were significantly deregulated between normal and tumor samples (Figure 1D), among which, four genes previously identified in the TCGA dataset: (1) *KCNN4* and *TRPM2* were again upregulated in tumor tissues with a 1.02 and 1.12-fold-change, respectively (Table S3). (2) *KCNMA1*, *TRPM6* and *TRPM4* were downregulated with a −1.13, −3.34 and −1.42 fold-change, respectively (Table S3). Indeed, *TRPM4*, which we found significantly down-regulated in tumor tissues (fold-change < −1) in the microarray dataset, was also down-regulated with a fold-change of −0.8 in the TCGA dataset (Table S2).

Given that the genes were able to discriminate between normal and tumor samples in both datasets, we next investigated the co-expression of Ca^2+^ and KCa channels and the possible correlation between their expression and the progression of CRC.

### 2.2. Co-Expression of Ca^2+^ and KCa Channels in CRC

**Figure 2:**
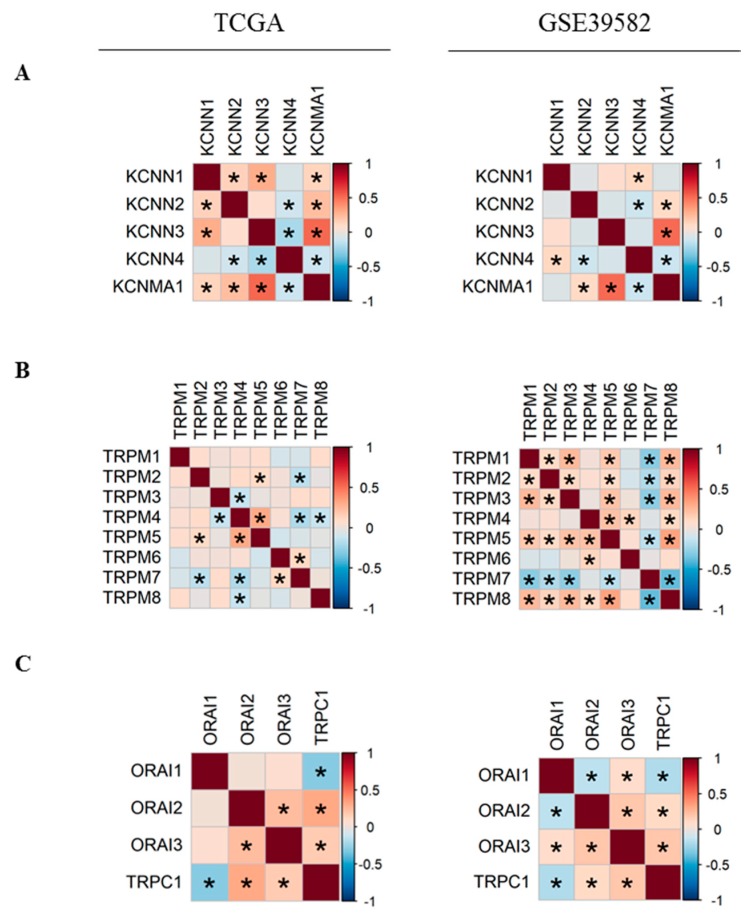
Pearson’s correlation heatmaps of gene expression in TCGA and GSE39582 datasets. (**A**) Pearson’s correlation heatmaps of KCa channels family coding genes; (**B**) Pearson’s correlation heatmaps of *TRPM* channels family coding genes; (**C**) Pearson’s correlation heatmaps of *ORAI* family and *TRPC1* genes. Pearson’s correlation coefficients were shown with continuous gradient colors. Red represents positive correlation whereas blue represents negative correlation. Asterisk symbol correspond to correlations with BH-adjusted *p*-value < 0.05. TCGA: The Cancer Genome Atlas; KCa: Ca^2+^-dependent potassium channels. BH: Benjamini-Hochberg.

In order to better understand the relationship between Ca^2+^ and KCa channels coding-genes, we analyzed their pairwise co-expressions by performing Pearson’s correlation analysis. A global matrix summarizing all pairwise correlations is presented in Supplementary Figure S1. Hierarchical clustering resulted in two clusters of positively-correlated genes in each dataset (Figure S1), suggesting the existence of possible co-regulation at the transcriptional level by common transcription factors. Since two KCa and two TRPM channels were differentially expressed between normal and tumor tissues, we decided to study more closely the co-expression of members within each of KCa and TRPM channel families (Figure 2). We found that the expression of *KCNMA1* is positively correlated with *KCNN2* and *KCNN3* expression (Figure 2A). In addition, *KCNN4* is negatively correlated with *KCNN2* and *KCNMA1* (Figure 2A). Moreover, we found that in both datasets, *KCNMA1* expression is positively correlated with the expression of both regulatory subunits *KCNMB1* and *KCNMB2* (Figure S1B). Moreover, *TRPM5* expression level is positively correlated with *TRPM2* and *TRPM4*, while *TRPM7* is negatively correlated with *TRPM2* and *TRPM5* in both datasets (Figure 2B). We also studied the co-expression of the most selective Ca^2+^ channels. We found that *ORAI2* and *ORAI3* are positively correlated (Figure 2C). *TRPC1* is positively correlated with both *ORAI2* and *ORAI3* and is negatively correlated with *ORAI1*. No correlation is found between the expression of *STIM1* and *STIM2* (Figure S1C).

### 2.3. Gene Expression Analysis According to Tumor Primary Site, Lymph Node Metastases and Tumor Stage

**Figure 3:**
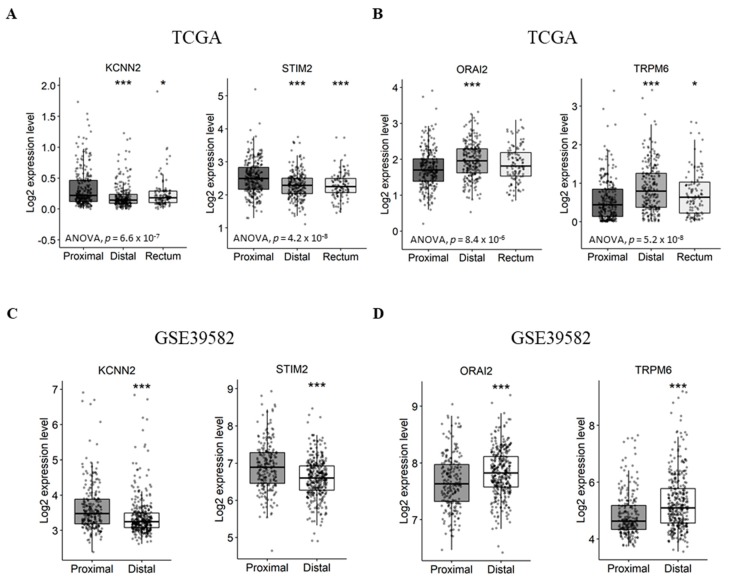
Differentially expressed genes between proximal and distal/rectal tumors in TCGA and GSE39582 datasets. (**A**) Boxplots of genes downregulated in distal/rectal tumors compared to proximal tumors in TCGA. (**B**) Boxplots of genes upregulated in distal/rectal tumors compared to proximal tumors in TCGA. (**C**) Boxplots of genes downregulated in distal tumors compared to proximal tumors in GSE39582. (**D**) Boxplots of genes upregulated in distal tumors compared to proximal tumors in GSE39582. ANOVA test was used for multiple comparisonsThe student’s *t*-test was used for pairwise comparison. (* *p* < 0.05; *** *p* < 0.001). ANOVA: analysis of variance; TCGA: The Cancer Genome Atlas.

First, we aimed to verify if the expression of Ca^2+^ and KCa channel coding-genes was correlated with tumor primary sites. In this way we compared gene expression levels between distal/rectal tumors and proximal tumors. Interestingly, we found that *STIM2* and *KCNN2* are more expressed in proximal tumors compared to distal tumors, both in the TCGA dataset (Figure 3A and Table S4. *STIM2*: fold-change −0.22 with *p* ≤ 0.001; *KCNN2*: fold-change −0.13 with *p* < 0.001) and the GSE39582 dataset (Figure 3C and Figure S5. *STIM2*: fold-change −0.31 with *p* < 0.001; *KCNN2*: fold-change −0.25 with *p* < 0.001). On the other hand, the results show that *ORAI2* and *TRPM6* are less expressed in proximal tumors compared to distal tumors, both, in the TCGA dataset (Figure 3B and Figure S4. *ORAI2*: fold-change 0.23 with *p* < 0.001; *TRPM6*: fold-change 0.31 with *p* < 0.001) and GSE39582 dataset (Figure 3D and Table S5. *ORAI2*: fold-change 0.16 with *p* < 0.001; *TRPM6*: fold-change 0.44 with *p* < 0.001). However, only the TCGA dataset contained rectum tumor samples, in which the expression profiles of *KCNN2*, *STIM2* and *TRPM6* followed that of the distal tumor samples (Figure 3A).

**Figure 4:**
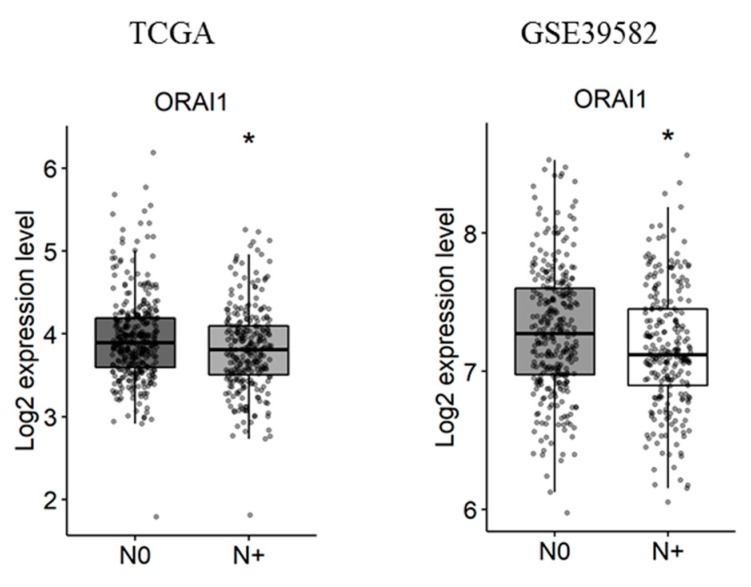
*ORAI1* is downregulated with lymph node metastases in TCGA and GSE39582 datasets. N0 represents tumors with no lymph node metastases and N+ represents tumors with N1 or N2 lymph node metastases. The student’s *t*-test was used for pairwise comparison. (* *p* < 0.05). TCGA: The Cancer Genome Atlas.

Next, the correlation of Ca^2+^ and KCa channels coding-gene expressions with lymph node metastases was studied. We found that, among the studied genes, only *ORAI1* is deregulated in tumors with lymph node metastases in both the TCGA and the GSE39582 datasets (Figure 4 and Tables S6 and S7. fold-change of −0.13 and 0.13 with *p* = 0.016 and *p* = 0.03, respectively).

Afterwards, we investigated the correlation of gene expression with CRC progression. For this purpose, we decided to compare gene expression in stage IV, the most aggressive stage with distant metastases, to that of stages I, II and III combined which are less advanced. Unfortunately, none of the genes were concomitantly deregulated in the two datasets (Tables S8 and S9).

### 2.4. ORAI3 and TRPC1 Expression Predict Poor Prognosis of CRC Patients

**Figure 5:**
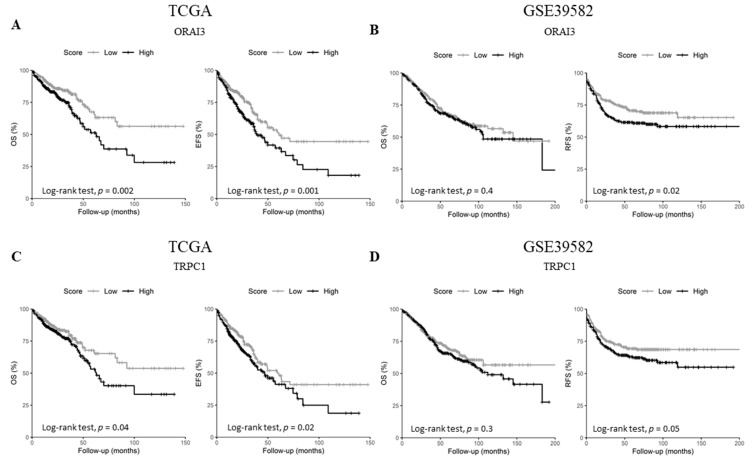
Kaplan-Meier survival curves of *ORAI3* and *TRPC1* genes in TCGA and GSE39582. (**A**) OS and EFS survival curves for *ORAI3* in the TCGA dataset; (**B**) OS and RFS survival curves for *ORAI3* in the GSE39582 dataset; (**C**) OS and EFS survival curves for *TRPC1* in the TCGA dataset; (**D**) OS and RFS survival curves for *ORAI3* in the GSE39582 dataset. High and Low scores were attributed to patients using gene-median expression as a threshold. Comparison between High and Low categories was done using a Log-Rank test. Abbreviations: OS: Overall survival. EFS: Event-free survival. RFS: Relapse-free survival.

Since the expression of the selected genes did not correlate with tumor stage, we questioned whether they could harbor some prognostic information. Therefore, we checked the genes’ prognostic values on overall survival (OS) in both datasets, in addition to event-free survival (EFS) and relapse-free survival (RFS) for either TCGA or GSE39582 datasets, respectively. Univariate Cox regression analysis showed that *ORAI3* and *TRPC1* are the only genes that are concomitantly prognostic factors in both datasets (Table 1 and Tables S10 and S11). In fact, *ORAI3* correlated with a poor outcome for both OS and EFS in the TCGA dataset (Table 1. OS HR = 1.72 with *p* = 0.003 and EFS HR = 1.6 with *p* = 0.001; and Figure 5A. OS Log-Rank *p* = 0.002 and EFS Log-Rank *p* = 0.001) whereas it only correlated with RFS in the GSE39582 dataset (Table 1. RFS HR = 1.6 with *p* = 0.001; and Figure 5B. RFS Log-Rank *p* = 0.02). Likewise, *TRPC1* was correlated with poor outcome for OS and EFS in the TCGA dataset (Table 1. OS HR = 1.44 with *p* = 0.042 and EFS HR = 1.39 with *p* = 0.025; and Figure 5C. OS Log-Rank *p* = 0.04 and EFS Log-Rank *p* = 0.02), whereas it was only correlated with RFS in the GSE38582 dataset (Table 1. RFS HR = 1.43 with *p* = 0.05; and Figure 5D. RFS Log-Rank *p* = 0.05).

### 2.5. Upregulated ORAI3/ORAI1 Ratio Predicts Poor Prognosis of CRC Patients

**Figure 6:**
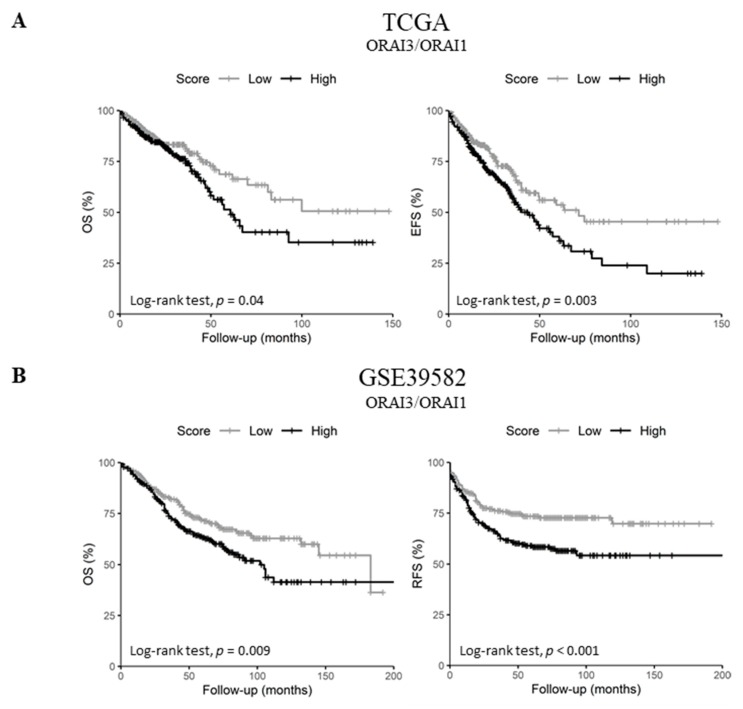

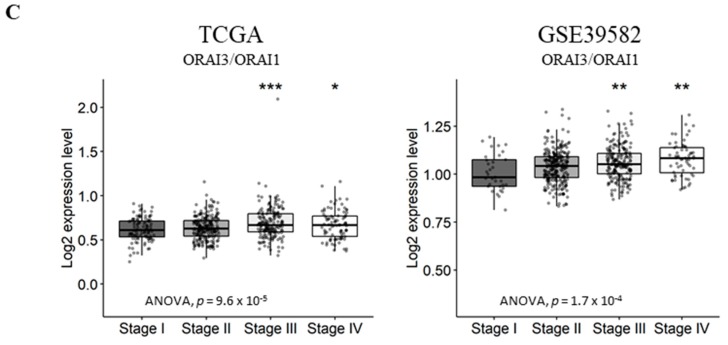
*ORAI3/ORAI1* ratio predicts poor prognosis in CRC. High and Low scores were attributed to patients using ratio *ORAI3/ORAI1* median expression as a threshold. High and Low categories were done using a Log-Rank test; (**A**) OS and EFS survival curves of *ORAI3/ORAI1* ratio in the TCGA dataset. (**B**) OS and RFS survival curves of *ORAI3/ORAI1* ratio in the GSE39582; (**C**) *ORAI3/ORAI1* ratio expression according to tumor stages. The ANOVA test was used for multiple comparisons. The BH-adjusted student *t*-test was used for pairwise comparisons (Stage I was considered a reference). (* *p* < 0.05; ** *p* < 0.01; *** *p* < 0.001).

Since *ORAI1* expression decreased in lymph node metastatic tumors and high *ORAI3* expression was a predictor of poor prognosis in both datasets, we investigated the possibility of a functional shift in the Ca^2+^ entry from SOCE (ORAI1-dependent) to Store-independent Ca^2+^ channel entry SICE (ORAI3-dependent) and its association with a poor outcome, as described in prostate and breast cancers [23,24]. Therefore, we investigated whether the ratio of *ORAI3/ORAI1* is correlated with the survival of CRC patients. Indeed, Pearson’s correlation showed that *ORAI1* and *ORAI3* expression levels are poorly correlated in both datasets (Figure 2C and Figure S1D. R = 0.07 with *p* = 0.095 for the TCGA dataset and R = 0.09 with *p* = 0.032 for GSE39582 dataset). However, despite the lack of apparent correlation, we found that a high *ORAI3/ORAI1* ratio correlated with a poor outcome for OS and EFS in the TCGA dataset (Table 2. OS HR = 1.45 with *p* = 0.037 and EFS HR = 1.54 with *p* = 0.003; and Figure 6A. OS Log-Rank *p* = 0.04 and EFS Log-Rank *p* = 0.003). In addition, this ratio also correlated with a poor outcome for OS and RFS in the GSE39582 dataset (Table 2. OS HR = 1.47 with *p* = 0.009 and RFS HR = 1.69 with *p* = 0.001; and Figure 6B. OS Log-Rank *p* = 0.009 and RFS Log-Rank *p* < 0.001).

Next, we investigated the correlation of *ORAI3/ORAI1* ratio with CRC progression by studying its variation according to tumor stages. Interestingly, although the genes individually did not correlate with tumor progression (Tables S8 and S9), the *ORAI3/ORAI1* Ratio was significantly increased in stages III and IV compared to Stage I in both TCGA and GSE39582 datasets (Figure 6C). Taken together, those results indicate that a Ca^2+^ influx switch could exist between *ORAI3* and *ORAI1* expressions in favor of the former during the progression of CRC, explaining its association with a poor outcome.

## 3. Discussion

Several studies investigated the role of ion channels in cancer, providing evidence of their important roles in cell proliferation, migration, apoptosis and differentiation, and thus the designation “onco-channels” [25,26]. In this study, we investigated the transcriptional expression profile of Ca^2+^ and KCa channels in CRC by exploring two expression datasets: RNAseq (TCGA) and microarray (GSE39582). Those two datasets were selected because they are both large (*n* > 500), diverse (contain normal and CRC samples of various stages) and annotated for patients’ survival beside other clinical information. Although many genes gave significant results in one of the two datasets individually, we only focused our analysis on robust results that were constantly significant in both datasets.

The expression of the selected Ca^2+^ and KCa channels coding genes discriminated between tumor and normal tissues. Four consistent genes were differentially expressed in tumor tissues compared to normal tissues: two Ca^2+^-activated channels coding genes were induced (*KCNN4*, *TRPM2*) while *KCNMA1* and *TRPM6* were downregulated during the malignant transformation. The decrease in *KCNMA1* expression level is in agreement with observations showing that it is downregulated in CRC patients through epigenetic and microRNA regulations [27]. Nonetheless, a murine study with the adenomatous polyposis coli (APC) mutation model had shown increased *KCNMB1* and *KCNMB2* expression, suggesting a possible increase of KCNMA1 activity in CRC initiation [28].

Likewise, increased expression of *KCNN4* was reported in various cancers [29]. Taken together, those results propose a switch in the expression of Ca^2+^ and voltage activated potassium channels (*KCNMA1*) to Ca^2+^ and non-voltage activated potassium channels (*KCNN4*) in CRC. Indeed, a similar switch was reported in restenosis leading to an increased proliferation and migration of smooth muscle cells [30]. Interestingly, SK4 was found in the inner mitochondrial membrane of human CRC cells where it may control Ca^2+^ signaling [31]. Besides, SK4 was found to induce epithelial mesenchymal transition in CRC cell lines in vitro and was suggested to promote cancer metastasis [32].

Our results showed that *TRPM6* is decreased in CRC cells in contrary to *TRPM2* and remarkably, we also found that *TRPM2* is negatively correlated with *TRMP7*. Indeed, TRPM6 and TRPM7 are implicated in Mg^2+^ uptake in normal epithelial colon cells [33]. Besides, colon cancer cells that are resistant to doxorubicin expressed lower amounts of TRPM6 and TRPM7 [34]. Those results suggest that the Mg^2+^ homeostasis could be lost during the malignant transformation of CRC. On the other hand, TRPM2 is known to promote cell survival and protect against oxidative stress in various cell types [35]. It is also highly expressed in many solid cancers such as pancreatic, melanoma, neuroblastoma, breast and prostate cancer [35]. Interestingly, TRPM2 was found to be activated by cytosolic adenosine diphosphate ribose (ADP-ribose) and cytosolic Ca^2+^ [36,37] contrarily to TRPM6 that is only activated by cytosolic Mg^2+^ depletion. Moreover, the activation of TRPM2 induces the expression of transcription factors such as hypoxia-inducible factor 1 alpha (HIF1α) and nuclear factor (erythroid-derived 2)-related factor-2 (Nrf2) [35]. These factors are implicated in cell proliferation, survival and chemoresistance of CRC cells [38,39]. Our results, together with those findings, suggest that Ca^2+^-activated (SK4) and Ca^2+^-permeable channels (TRPM2) could be induced in CRC malignant transformations.

Colorectal carcinoma localization is associated with differences in key molecular features and clinical implications. Generally, patients with proximal colon cancers have a worse prognosis [40], whereas only patients with metastases from a distal carcinoma respond to anti- epithelial growth factor receptor (anti-EGFR) therapy [41]. Our results showed that *STIM2* and *KCNN2* are upregulated while *ORAI2* and *TRPM6* are downregulated in proximal tumors. The variation of Ca^2+^ and KCa channels gene expression according to tumor primary site suggests their implication in CRC biology and heterogeneity.

The Villalobos group found that normal mucosa cells showed an I_CRAC_ current mediated by ORAI1. Conversely, colon carcinoma cells showed mixed currents composed of enhanced I_CRAC_ besides a nonselective Ca^2+^ current mediated by TRPC1 [17]. Moreover, the expression of TRPC1, ORAI1, ORAI2, ORAI3 and STIM1 was increased in tumor cells in contrary to STIM2 protein which was found to be depleted [17]. Our study showed that *TRPC1* is negatively correlated with *ORAI1* and positively correlated with *ORAI2* and *ORAI3*, and that *TRPC1* expression predicted a poor prognosis in CRC. This result suggests a SOCE remodeling during CRC progression and could be the subject of further analysis.

Our results show that *ORAI1* decreased with lymph node metastases, one of the first signs of metastatic spread. Also, *ORAI3* predicted a poor prognosis for RFS in GSE39582 but not for OS, and *ORAI1* predicted poor prognosis in TCGA but not in GSE39582 (Table S10). However, by inspecting the ratio of *ORAI3/ORAI1*, we found that a high *ORAI3/ORAI1* ratio predicted a poor prognosis for EFS and OS in TCGA, as well as for RFS and EFS in GSE39582, with a higher HR than that of *ORAI1* or *ORAI3* separately. Thus, the *ORAI3/ORAI1* ratio is a better predictor of survival than *ORAI3* alone. This finding is consistent with two other studies that described a change in Ca^2+^ influx from ORAI1 to ORAI3 in cancer. In prostate cancer, it was suggested that *ORAI1* is implicated in a dynamic machinery of Ca^2+^ remodeling between *ORAI1* and *ORAI3* [23]. It was postulated that ORAI3 increases limits SOCE and enhances ORAI1-ORAI3 ARC Ca^2+^ entry [23]. Moreover, high *ORAI3/ORAI1* ratio was shown to correlate with poor prognosis in breast cancer [24]. However, this ratio should not undermine the pro-tumor role of ORAI1. Indeed, we have previously shown that ORAI1 contributes to CRC cell migration [20], and significantly, it was found that *ORAI1* expression is increased in CRC tissues compared to adjacent non-cancerous tissues and is correlated with distant metastases [42].

To our knowledge, our study is the first to widely analyze Ca^2+^ and Ca^2+^-activated K^+^ channels remodeling in CRC using high throughput data analysis. Our results provide new insights about the voltage gating of KCa channels and Ca^2+^ remodeling in CRC initiation and progression.

## 4. Materials and Methods

### 4.1. Public Datasets

Two CRC datasets, available from public databases, were used in this paper: an RNAseq dataset (TCGA, *n* = 656 including 47 normal samples) and an expression microarray dataset (GSE39582, *n* = 585 including 19 normal samples) [43]. The two datasets were selected because of their large size, diversity and the availability of clinical annotations. For the RNAseq dataset, Fragment Per Kilobase Million (FPKM) values were downloaded from The Cancer Genome Atlas (TCGA) database (https://portal.gdc.cancer.gov/repository) [44] and were log2 transformed after the addition of one (log2(x + 1)) prior to analysis. Clinical annotations were acquired from cbioportal for Cancer Genomics website (http://cbioportal.org) using the the Cancer Genomics Data Server package (cgdsr) in R environment [45,46]. GSE39582 microarray dataset (RMA-normalized, Affimetrix Human Genome U133 Plus 2.0 Array) was downloaded from the Gene Expression Omnibus (GEO) database (https://www.ncbi.nlm.nih.gov/geo/). For each gene, the probeset with the average expression was used. Thirty-five genes related to Ca^2+^ and KCa channels were selected for analysis when available (TRPV1 was missing from the microarray dataset). Gene names and functions are summarized in Supplementary Table S1.

### 4.2. Statistical Analysis

Heatmaps Boxplots and correlation plots were generated in R using ComplexHeatmap [47], ggpubr (https://CRAN.R-project.org/package=ggpubr) and corrplot (https://github.com/taiyun/corrplot) packages, respectively. Hierarchical clustering was done using Euclidean distance and average linkage method for both genes and samples. Pearson’s test was used for gene correlation analysis. Wilcoxon’s signed-rank test was used to compare matched normal and tumor tissues in the TCGA dataset. A student’s *t*-test was used for the other pairwise comparisons in the paper. The Benjamini and Hochberg method was used to adjust for multiple comparisons [48], and adjusted *p*-values below 0.05 were considered significant.

### 4.3. Survival Analysis

Overall survival (OS) was defined by the time from CRC diagnosis (in months) until death (patients alive at last follow-up were censored). Event-free survival (EFS) was defined as the time from diagnosis until the occurrence of an event (progression, recurrence or death), and patients with no reported events were censored at last follow-up. For patients achieving complete remission, relapse-free survival (RFS) was defined as the time from surgery to the first recurrence (Patients with no recurrence were censored upon death or last follow-up).

For each gene, High and Low expression scores were assigned to patients using median expression as cut-off threshold. For the *ORAI3/ORAI1* ratio, High and Low scores were assigned using the median ratio as cut-off threshold. Cox proportional hazard regression was used to perform dichotomic univariate analyses. Wald’s test was used to estimate prognostic significance of model’s parameters. Schoenfeld residuals were used to check for violation of proportional hazards assumption [49]. Kaplan-Meier survival curves were used to plot High and Low score groups for prognostic genes. Groups were compared using the Log-Rank test. Survival analyses were done in R environment using survival and survminer packages (https://CRAN.R-project.org/package=survival, https://CRAN.R-project.org/package=survminer).

## 5. Conclusions

In summary, we found that KCa and Ca^2+^ channels could be important contributors to CRC initiation. Our results suggested a switch from voltage gating in KCa channels (*KCNMA1*) to two different types of channels activated by cytosolic Ca^2+^ and voltage-insensitive (*KCNN4*, *TRPM2*) during the malignant transformation. We also showed that KCa and Ca^2+^ channels are differentially expressed according to tumor site. Most of the literature focused on the role of ORAI1 in CRC. However, our results highlighted that *ORAI3/ORAI1* ratio predicts poor prognosis in CRC patients and could be considered as biomarker for Ca^2+^ remodeling during the CRC progression. Hence, future studies about ORAI3 are needed to understand the switch from SOCE influx to SICE influx in CRC progression and aggressiveness.

## Figures and Tables

**Table 1:** Univariate analysis of genes correlated with survival in TCGA and GSE39582.

Variable	Hazard Ratio	95% CI	Wald’s *p*-Value
**TCGA (OS *n* = 602)**			
ORAI3 (High vs. Low)	1.72	(1.21–2.45)	0.003
TRPC1 (High vs. Low)	1.44	(1.01–2.06)	0.042
**TCGA (EFS *n* = 602)**			
ORAI3 (High vs. Low)	1.6	(1.2–2.13)	0.001
TRPC1 (High vs. Low)	1.39	(1.04–1.85)	0.025
**GSE39582 (OS *n* = 562)**			
ORAI3 (High vs. Low)	1.12	(0.84–1.48)	0.443
TRPC1 (High vs. Low)	1.18	(0.89–1.57)	0.258
**GSE39582 (RFS *n* = 557)**			
ORAI3 (High vs. Low)	1.43	(1.06–1.92)	0.02
TRPC1 (High vs. Low)	1.34	(1–1.81)	0.05

Abbreviations: OS: Overall survival. EFS: Event-free survival. RFS: Relapse-free survival. CI: confidence interval.

**Table 2:** Univariate analysis of *ORAI3/ORAI1* ratio with survival in TCGA and GSE39582.

Variable	Hazard Ratio	95% CI	Wald’s *p*-Value
**TCGA (OS *n* = 602)**			
ORAI3/1 ratio (High vs. Low)	1.45	(1.02–2.07)	0.037
**TCGA (EFS *n* = 602)**			
ORAI3/1 ratio (High vs. Low)	1.54	(1.15–2.06)	0.003
**GSE39582 (OS *n* = 562)**			
ORAI3/1 ratio (High vs. Low)	1.47	(1.1–1.95)	0.009
**GSE39582 (RFS *n* = 557)**			
ORAI3/1 ratio (High vs. Low)	1.69	(1.25–2.28)	0.001

Abbreviations: OS: Overall survival. EFS: Event-free survival. RFS: Relapse-free survival. CI: confidence interval.

## Supplementary Materials

The following are available online at https://www.mdpi.com/2072-6694/11/4/561/s1, Figure S1: Pearson’s correlation of gene expression in TCGA and GSE39582 datasets. (A) Pearson’s correlation heatmaps of Ca^2+^ and KCa channels family coding genes; (B) Scatter plots of KCNMA1 and KCNMB subunits correlation; (C) Scatter plots of STIM1 and STIM2 correlation; (D) Scatter plots of ORAI1 and ORAI3 correlation, Table S1: Symbols, names and functions of the selected genes, Table S2: Comparison of gene expression levels in normal mucosa versus tumor samples in TCGA dataset, Table S3: Comparison of gene expression levels in normal mucosa versus tumor samples in GSE39582, Table S4: Comparison of gene expression levels between proximal and distal/rectal tumors in TCGA dataset, Table S5: Comparison of gene expression levels between proximal and distal tumors in GSE39582 dataset, Table S6: Comparison of gene expression levels between N0 and N+ (lymph node metastatic) tumors in TCGA dataset, Table S7: Comparison of gene expression levels between N0 and N+ (lymph node metastatic) tumors in GSE39582 dataset, Table S8: Comparison of gene expression levels between stage IV and stages I + II + III in TCGA dataset, Table S9: Comparison of gene expression levels between stage IV and stages I + II + III in GSE39582 dataset, Table S10: Univariate analysis of OS and EFS in TCGA dataset, Table S11: Univariate analysis of OS and RFS in GSE39582 dataset.
