# Supplementary material for: Expression Profiling of Calcium Channels and Calcium-Activated Potassium Channels in Colorectal Cancer
Source: Cancers (Basel). 2019 Apr 19;11(4):561. doi: 10.3390/cancers11040561 (PMC6521016; doi:10.3390/cancers11040561)
Supplement: Supplementary file 1 [file cancers-11-00561-s001.pdf]

Supplementary Material: Expression Profiling of Calcium Channels and Calcium-Activated Potassium Channels in Colorectal Cancer

Sajida Ibrahim, Hassan Dakik, Christophe Vandier, Romain Chautard, Gilles Paintaud, Frédéric Mazurier, Thierry Lecomte, Maxime Guéguinou and William Raoul

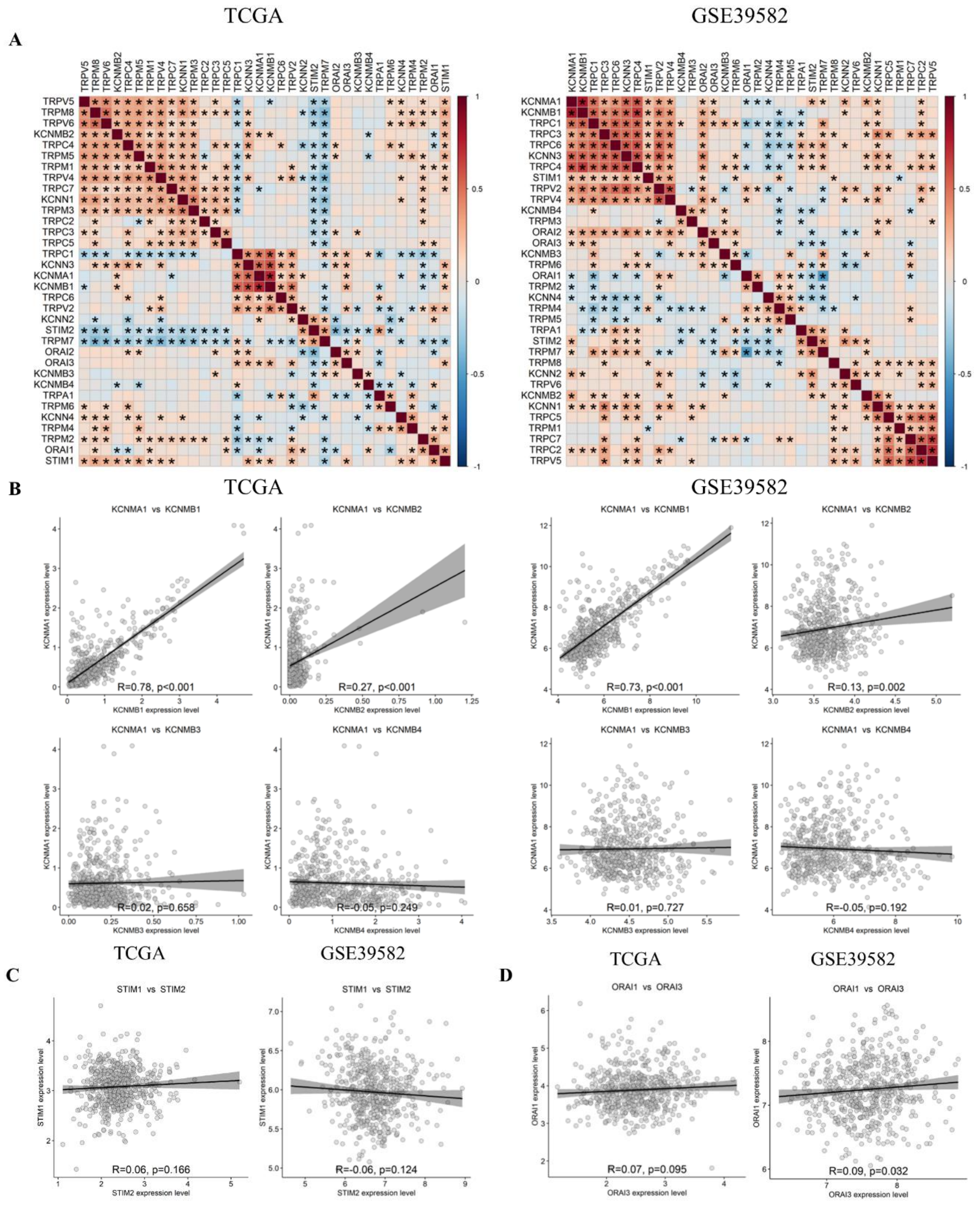

Figure S1. Pearson's correlation of gene expression in TCGA and GSE39582 datasets. (**A**) Pearson's correlation heatmaps of Ca^{2+} and KCa channels family coding genes; (**B**) Scatter plots of KCNMA1 and KCNMB subunits correlation; (**C**) Scatter plots of STIM1 and STIM2 correlation; (**D**) Scatter plots

of ORAI1 and ORAI3 correlation. Asterisk symbol correspond to correlations with BH-adjusted *p*-value < 0.05.

Table S1. Symbols, names and functions of the selected genes.

Gene Symbol	Gene Name	Function/Channel Type
KCNN1	Small conductance potassium calcium-activated channel subfamily N member 1	calcium-activated potassium channel
KCNN2	Small conductance potassium calcium-activated channel subfamily N member 2	calcium-activated potassium channel
KCNN3	Small conductance potassium calcium-activated channel subfamily N member 3	calcium-activated potassium channel
KCNN4	Intermediate conductance potassium calcium-activated channel subfamily N member 4	voltage-independent potassium channel activated by intracellular calcium
KCNMA1	Big conductance Potassium Calcium-Activated Channel Subfamily M Alpha 1	voltage-dependent potassium channel activated by intracellular calcium
KCNMB1	Potassium Calcium-Activated Channel Subfamily M Regulatory Beta Subunit 1	Calcium-Activated Channel Subfamily M Regulatory Beta Subunit
KCNMB2	Potassium Calcium-Activated Channel Subfamily M Regulatory Beta Subunit 2	Potassium Calcium-Activated Channel Subfamily M Regulatory Beta Subunit
KCNMB3	Potassium Calcium-Activated Channel Subfamily M Regulatory Beta Subunit 3	Potassium Calcium-Activated Channel Subfamily M Regulatory Beta Subunit
KCNMB4	Potassium Calcium-Activated Channel Subfamily M Regulatory Beta Subunit 4	Potassium Calcium-Activated Channel Subfamily M Regulatory Beta Subunit
STIM1	Stromal interaction molecule 1	Ca2+ sencor controlling the entry of calcium into cells
STIM2	Stromal interaction molecule 2	Ca2+ sencor controlling the entry of calcium into cells
ORAI1	Calcium release-activated calcium modulator 1	Calcium release-activated Ca2+ (CRAC) channel subunit
ORAI2	Calcium release-activated calcium modulator 2	Calcium release-activated Ca2+ (CRAC) channel subunit
ORAI3	Calcium release-activated calcium modulator 3	Arachidonate Regulated Ca2+ (ARC) channel
TRPA1	Transient receptor potential cation channel subfamily A member 1	Transient receptor potential cation channel
TRPC1	Transient receptor potential cation channel subfamily C member 1	Transient receptor potential cation channel
TRPC2	Transient receptor potential cation channel subfamily C member 2	Transient receptor potential cation channel
TRPC3	Transient receptor potential cation channel subfamily C member 3	Transient receptor potential cation channel
TRPC4	Transient receptor potential cation channel subfamily C member 4	Transient receptor potential cation channel
TRPC5	Transient receptor potential cation channel subfamily C member 5	Transient receptor potential cation channel
TRPC6	Transient receptor potential cation channel subfamily C member 6	Transient receptor potential cation channel
TRPC7	Transient receptor potential cation channel subfamily C member 7	Transient receptor potential cation channel
TRPV1	Transient receptor potential cation channel subfamily V member 1	Transient receptor potential cation channel
TRPV2	Transient receptor potential cation channel subfamily V member 2	Transient receptor potential cation channel
TRPV4	Transient receptor potential cation channel subfamily V member 3	Transient receptor potential cation channel
TRPV5	Transient receptor potential cation channel subfamily V member 4	Transient receptor potential cation channel
TRPV6	Transient receptor potential cation channel subfamily V member 5	Transient receptor potential cation channel
TRPM1	Transient receptor potential cation channel subfamily M member 1	Transient receptor potential cation channel
TRPM2	Transient receptor potential cation channel subfamily M member 2	Transient receptor potential cation channel

TRPM3	Transient receptor potential cation channel subfamily M member 3	Transient receptor potential cation channel
TRPM4	Transient receptor potential cation channel subfamily M member 4	Transient receptor potential cation channel
TRPM5	Transient receptor potential cation channel subfamily M member 5	Transient receptor potential cation channel
TRPM6	Transient receptor potential cation channel subfamily M member 6	Transient receptor potential cation channel
TRPM7	Transient receptor potential cation channel subfamily M member 7	Transient receptor potential cation channel
TRPM8	Transient receptor potential cation channel subfamily M member 8	Transient receptor potential cation channel

Table S2. Comparison of gene expression levels in normal mucosa versus tumor samples in TCGA dataset.

		TCGA		
**Gene**	**FC (Tumor vs. Normal)**	**95% CI**	**BH-Adjusted *p*-Value**	**Significance**
KCNN1	−0.04	(−0.05; −0.02)	<0.001	***
KCNN2	0	(−0.1; 0.1)	0.9300	
KCNN3	−0.72	(−0.89; −0.55)	<0.001	***
KCNN4	1.24	(0.81; 1.67)	<0.001	***
KCNMA1	−1.66	(−1.99; −1.34)	<0.001	***
KCNMB1	−1.73	(−2.18; −1.29)	<0.001	***
KCNMB2	−0.13	(−0.19; −0.08)	<0.001	***
KCNMB3	0.06	(0.03; 0.1)	<0.001	***
KCNMB4	−0.05	(−0.29; 0.19)	0.4200	
ORAI1	0.56	(0.36; 0.76)	<0.001	***
ORAI2	0.33	(0.16; 0.5)	0.0012	**
ORAI3	0.25	(0.12; 0.38)	0.0014	**
STIM1	−0.78	(−0.93; −0.62)	<0.001	***
STIM2	0.21	(0.06; 0.36)	0.0520	
TRPA1	−0.52	(−0.79; −0.26)	0.0012	**
TRPC1	−0.42	(−0.56; −0.29)	<0.001	***
TRPC2	−0.01	(−0.03; 0.01)	0.0110	*
TRPC3	−0.01	(−0.04; 0.02)	0.2600	
TRPC4	−0.13	(−0.23; −0.03)	0.0530	
TRPC5	0	(0; 0)	0.7600	
TRPC6	−0.32	(−0.41; −0.23)	<0.001	***
TRPC7	−0.14	(−0.17; −0.11)	<0.001	***
TRPV1	0	(−0.02; 0.01)	0.4100	
TRPV2	−0.01	(−0.24; 0.22)	0.7800	
TRPV4	0.58	(0.43; 0.73)	<0.001	***
TRPV5	0.01	(0; 0.02)	0.0560	
TRPV6	0.02	(−0.04; 0.08)	0.0520	
TRPM1	0	(0; 0.01)	0.2300	
TRPM2	1.28	(1.04; 1.51)	<0.001	***
TRPM3	0	(−0.01; 0.02)	0.2600	
TRPM4	−0.8	(−1.09; −0.51)	<0.001	***
TRPM5	−0.27	(−0.49; −0.05)	0.0110	*
TRPM6	−2.76	(−3.12; −2.4)	<0.001	***
TRPM7	−0.28	(−0.44; −0.11)	0.0054	**
TRPM8	0.03	(0.01; 0.05)	0.2000	

* $p < 0.05$; ** $p < 0.01$; *** $p < 0.001$.

.

Table S3. Comparison of gene expression levels in normal mucosa versus tumor samples in GSE39582 dataset.

GSE39582				
**Gene**	**FC (Tumor vs. Normal)**	**Mean (95% CI)**	**BH-Adjusted *p*-Value**	**Significance**
KCNN1	0.06	5.11 (5.09; 5.13)	0.31	
KCNN2	0.09	3.5 (3.44; 3.55)	0.9	
KCNN3	0.35	4.82 (4.77; 4.86)	0.002	**
KCNN4	1.02	7.99 (7.91; 8.07)	<0.001	***
KCNMA1	−1.13	6.93 (6.83; 7.04)	<0.001	***
KCNMB1	−0.1	5.79 (5.7; 5.88)	0.71	
KCNMB2	−0.12	3.65 (3.63; 3.67)	0.015	*
KCNMB3	−0.02	4.47 (4.44; 4.5)	0.66	
KCNMB4	0.87	6.02 (5.94; 6.11)	<0.001	***
ORAI1	0.74	7.25 (7.21; 7.28)	<0.001	***
ORAI2	−0.06	7.77 (7.73; 7.8)	0.55	
ORAI3	0.48	7.57 (7.53; 7.6)	<0.001	***
STIM1	−0.23	5.97 (5.94; 6)	0.0062	**
STIM2	−0.15	6.72 (6.67; 6.77)	0.14	
TRPA1	−0.95	6.12 (6.03; 6.21)	<0.001	***
TRPC1	0.05	4.61 (4.56; 4.67)	0.73	
TRPC2	−0.15	3.81 (3.79; 3.83)	0.0059	**
TRPC3	−0.03	2.91 (2.9; 2.92)	0.39	
TRPC4	0.19	5.14 (5.12; 5.16)	0.0036	**
TRPC5	0	2.65 (2.64; 2.66)	0.9	
TRPC6	0.13	3.7 (3.68; 3.73)	0.057	
TRPC7	0.08	3.51 (3.49; 3.53)	0.15	
TRPM1	0.13	2.89 (2.87; 2.9)	0.0061	**
TRPM2	1.12	6.37 (6.32; 6.42)	<0.001	***
TRPM3	−0.05	4.16 (4.14; 4.18)	0.55	
TRPM4	−1.42	7.5 (7.43; 7.57)	<0.001	***
TRPM5	0.08	4.11 (4.08; 4.13)	0.55	
TRPM6	−3.34	5.12 (5.04; 5.19)	<0.001	***
TRPM7	−0.52	6.33 (6.28; 6.39)	<0.001	***
TRPM8	−0.18	4.65 (4.63; 4.67)	0.0025	**
TRPV2	0.51	6.14 (6.1; 6.19)	<0.001	***
TRPV4	0.34	5.5 (5.47; 5.53)	<0.001	***
TRPV5	0.02	4.84 (4.82; 4.86)	0.71	
TRPV6	−0.01	5.74 (5.72; 5.77)	0.67	

* $p < 0.05$; ** $p < 0.01$; *** $p < 0.001$.

Table S4. Comparison of gene expression levels between proximal and distal/rectal tumors in TCGA dataset.

TCGA					
**Gene**	**Primary Site**	**FC (vs. Proximal)**	***p*-Value**	**BH-Adjusted *p*-Value**	**Significance**
KCNN1	Distal	−0.03	0.0254	0.0998	
KCNN1	Rectum	−0.02	0.1084	0.2784	
KCNN2	Distal	−0.13	<0.001	<0.001	***
KCNN2	Rectum	−0.09	0.0032	0.0227	*
KCNN3	Distal	−0.03	0.1379	0.3115	
KCNN3	Rectum	−0.04	0.1193	0.2784	
KCNN4	Distal	0.07	0.5267	0.6963	
KCNN4	Rectum	0.11	0.3597	0.5473	
KCNMA1	Distal	−0.06	0.2708	0.4861	
KCNMA1	Rectum	−0.03	0.705	0.809	
KCNMB1	Distal	0.04	0.4647	0.661	
KCNMB1	Rectum	0.08	0.3527	0.5473	

KCNMB2	Distal	−0.01	0.0908	0.264	
KCNMB2	Rectum	−0.01	0.6311	0.7709	
KCNMB3	Distal	0.03	0.0157	0.069	
KCNMB3	Rectum	0.01	0.5659	0.722	
KCNMB4	Distal	0.14	0.0496	0.1826	
KCNMB4	Rectum	0.12	0.1966	0.4048	
ORAI1	Distal	−0.02	0.6042	0.7552	
ORAI1	Rectum	0.21	0.0013	0.0101	*
ORAI2	Distal	0.23	<0.001	<0.001	***
ORAI2	Rectum	0.13	0.0257	0.0998	
ORAI3	Distal	0.08	0.0656	0.2047	
ORAI3	Rectum	0.06	0.2815	0.4927	
STIM1	Distal	0.01	0.866	0.8785	
STIM1	Rectum	0.05	0.2647	0.4861	
STIM2	Distal	−0.22	<0.001	<0.001	***
STIM2	Rectum	−0.23	<0.001	<0.001	***
TRPA1	Distal	−0.01	0.8597	0.8785	
TRPA1	Rectum	−0.02	0.7724	0.8342	
TRPC1	Distal	0.05	0.0591	0.1991	
TRPC1	Rectum	0	0.982	0.982	
TRPC2	Distal	−0.01	0.0597	0.1991	
TRPC2	Rectum	−0.01	0.0943	0.264	
TRPC3	Distal	−0.01	0.1164	0.2784	
TRPC3	Rectum	−0.01	0.4249	0.6196	
TRPC4	Distal	0	0.7563	0.8342	
TRPC4	Rectum	−0.01	0.4721	0.661	
TRPC5	Distal	0	0.8188	0.8582	
TRPC5	Rectum	0	0.1674	0.3551	
TRPC6	Distal	−0.02	0.2235	0.4341	
TRPC6	Rectum	0.01	0.6388	0.7709	
TRPC7	Distal	−0.01	0.3163	0.5272	
TRPC7	Rectum	0	0.8214	0.8582	
TRPV1	Distal	0	0.3088	0.5272	
TRPV1	Rectum	0	0.3395	0.5473	
TRPV2	Distal	−0.19	0.0044	0.0237	*
TRPV2	Rectum	−0.03	0.6984	0.809	
TRPV4	Distal	0.02	0.6708	0.7958	
TRPV4	Rectum	0.07	0.2173	0.4341	
TRPV5	Distal	−0.01	0.0158	0.069	
TRPV5	Rectum	−0.01	0.004	0.0237	*
TRPV6	Distal	−0.17	<0.001	<0.001	***
TRPV6	Rectum	−0.17	<0.001	<0.001	***
TRPM1	Distal	−0.01	0.2295	0.4341	
TRPM1	Rectum	−0.01	0.0044	0.0237	*
TRPM2	Distal	−0.11	0.1621	0.3546	
TRPM2	Rectum	−0.06	0.5673	0.722	
TRPM3	Distal	0.01	0.5272	0.6963	
TRPM3	Rectum	−0.01	0.1123	0.2784	
TRPM4	Distal	−0.11	0.0983	0.2647	
TRPM4	Rectum	−0.02	0.7746	0.8342	
TRPM5	Distal	−0.09	0.0673	0.2047	
TRPM5	Rectum	−0.05	0.3845	0.5727	
TRPM6	Distal	0.31	<0.001	<0.001	***
TRPM6	Rectum	0.17	0.01	0.0498	*
TRPM7	Distal	0.01	0.7676	0.8342	
TRPM7	Rectum	−0.22	<0.001	<0.001	***
TRPM8	Distal	0.01	0.3564	0.5473	
TRPM8	Rectum	0.01	0.4832	0.6633	

* $p < 0.05$; *** $p < 0.001$.

Table S5. Comparison of gene expression levels between proximal and distal tumors in GSE39582 dataset.

		GSE39582		
**Gene**	**FC (Distal vs. Proximal)**	***p*-Value**	**BH-Adjusted *p*-Value**	**Significance**
KCNN1	0.02	0.384963	0.63	
KCNN2	−0.25	<0.001	<0.001	***
KCNN3	−0.02	0.676336	0.88	
KCNN4	0.04	0.656917	0.88	
KCNMA1	−0.1	0.346771	0.62	
KCNMB1	0	0.965678	0.99	
KCNMB2	0	0.989466	0.99	
KCNMB3	0.07	0.017718	0.086	
KCNMB4	0.1	0.253676	0.52	
ORAI1	−0.07	0.057673	0.19	
ORAI2	0.16	<0.001	<0.001	***
ORAI3	0.03	0.434135	0.64	
STIM1	−0.05	0.07477	0.21	
STIM2	−0.31	<0.001	<0.001	***
TRPA1	−0.01	0.897965	0.98	
TRPC1	0	0.965708	0.99	
TRPC2	0	0.84151	0.95	
TRPC3	0	0.751781	0.89	
TRPC4	0.02	0.261441	0.52	
TRPC5	0.01	0.205378	0.5	
TRPC6	−0.04	0.18652	0.49	
TRPC7	−0.03	0.220392	0.5	
TRPV1	NA	NA	NA	
TRPV2	−0.22	<0.001	<0.001	***
TRPV4	−0.06	0.062509	0.19	
TRPV5	0.01	0.711899	0.89	
TRPV6	0.01	0.759176	0.89	
TRPM1	−0.03	0.057591	0.19	
TRPM2	−0.12	0.012773	0.072	
TRPM3	0.02	0.334558	0.62	
TRPM4	0.06	0.412487	0.64	
TRPM5	0.01	0.673641	0.88	
TRPM6	0.44	<0.001	<0.001	***
TRPM7	−0.11	0.041214	0.18	
TRPM8	0.02	0.390846	0.63	

*** $p < 0.001$.

Table S6. Comparison of gene expression levels between N0 and N+ (lymph node metastatic) tumors in TCGA dataset.

		TCGA		
**Gene**	**FC (N+ vs. N0)**	***P*-Value**	**BH-Adjusted *P*-Value**	**Significance**
KCNN1	0.01	0.2654	0.404	
KCNN2	−0.03	0.1841	0.307	
KCNN3	0.04	0.0239	0.093	
KCNN4	0.03	0.7464	0.817	
KCNMA1	0.09	0.0788	0.197	
KCNMB1	0.19	0.0008	0.009	**
KCNMB2	0	0.8773	0.877	
KCNMB3	0.05	<0.001	<0.001	***
KCNMB4	0.07	0.3104	0.418	
ORAI1	−0.13	0.0028	0.016	*
ORAI2	0.09	0.0287	0.100	
ORAI3	0.14	0.0005	0.009	**
STIM1	0.01	0.794	0.817	

STIM2	−0.13	0.0011	0.009	**
TRPA1	−0.12	0.0439	0.140	
TRPC1	0.07	0.0068	0.034	*
TRPC2	0.01	0.0872	0.204	
TRPC3	0.01	0.0184	0.081	
TRPC4	0.04	0.0013	0.009	**
TRPC5	0	0.0725	0.195	
TRPC6	0.03	0.1363	0.244	
TRPC7	0.01	0.2054	0.327	
TRPV1	0	0.6384	0.798	
TRPV2	0.02	0.7765	0.817	
TRPV4	0.06	0.1395	0.244	
TRPV5	0.01	0.1207	0.244	
TRPV6	−0.05	0.0621	0.181	
TRPM1	0.01	0.1075	0.235	
TRPM2	−0.02	0.7571	0.817	
TRPM3	0	0.7851	0.817	
TRPM4	−0.07	0.2895	0.417	
TRPM5	0.07	0.1324	0.244	
TRPM6	0.05	0.2978	0.417	
TRPM7	−0.02	0.6938	0.817	
TRPM8	0	0.5655	0.733	

* $p < 0.05$; ** $p < 0.01$; *** $p < 0.001$.

Table S7. Comparison of gene expression levels between N0 and N+ (lymph node metastatic) tumors in GSE39582 dataset.

GSE39582				
**Gene**	**FC (N+ vs. N0)**	***p*-Value**	**BH-Adjusted *p*-Value**	**Significance**
KCNN1	−0.01	0.490441	0.73	
KCNN2	−0.05	0.368378	0.6	
KCNN3	0.06	0.26343	0.45	
KCNN4	−0.01	0.859186	0.91	
KCNMA1	0.17	0.107263	0.26	
KCNMB1	0.22	0.021122	0.11	
KCNMB2	0.03	0.103412	0.26	
KCNMB3	0.07	0.014825	0.11	
KCNMB4	−0.14	0.096933	0.26	
ORAI1	−0.13	<0.001	0.03	*
ORAI2	0.09	0.022255	0.11	
ORAI3	0.05	0.197395	0.39	
STIM1	0	0.914583	0.91	
STIM2	−0.11	0.029682	0.13	
TRPA1	−0.16	0.090975	0.26	
TRPC1	0.11	0.043021	0.15	
TRPC2	−0.04	0.016524	0.11	
TRPC3	0.01	0.525957	0.75	
TRPC4	0.05	0.014064	0.11	
TRPC5	0	0.912571	0.91	
TRPC6	0	0.8876	0.91	
TRPC7	0	0.835335	0.91	
TRPV1	NA	NA	NA	
TRPV2	0.06	0.219908	0.4	
TRPV4	0.06	0.041575	0.15	
TRPV5	0.02	0.39655	0.61	
TRPV6	−0.04	0.187785	0.39	
TRPM1	−0.01	0.681481	0.84	
TRPM2	−0.06	0.223887	0.4	
TRPM3	−0.01	0.746898	0.88	

TRPM4	−0.04	0.614779	0.8
TRPM5	0.04	0.156491	0.35
TRPM6	0.22	0.010322	0.11
TRPM7	−0.02	0.693706	0.84
TRPM8	0.01	0.593808	0.8

* $p < 0.05$.

Table S8. Comparison of gene expression levels between stage IV and stages I+II+III in TCGA dataset.

TCGA				
**Gene**	**FC (IV vs. I–III)**	***p*-Value**	**BH-Adjusted *p*-Value**	**Significance**
KCNN1	0.02	0.4717	0.7892	
KCNN2	−0.03	0.388	0.7694	
KCNN3	0	0.9213	0.9658	
KCNN4	0.13	0.294	0.767	
KCNMA1	−0.05	0.3631	0.7694	
KCNMB1	0.08	0.2612	0.767	
KCNMB2	0.01	0.6104	0.8185	
KCNMB3	0.04	0.0253	0.2952	
KCNMB4	0.11	0.2097	0.767	
ORAI1	−0.03	0.6708	0.8185	
ORAI2	0.07	0.2808	0.767	
ORAI3	0.06	0.3068	0.767	
STIM1	0.13	0.0088	0.154	
STIM2	−0.2	<0.001	0.014	*
TRPA1	−0.04	0.6782	0.8185	
TRPC1	0.03	0.4177	0.7694	
TRPC2	0	0.9658	0.9658	
TRPC3	0.01	0.343	0.7694	
TRPC4	0.01	0.6534	0.8185	
TRPC5	0	0.2787	0.767	
TRPC6	0	0.8982	0.9658	
TRPC7	0	0.8402	0.9658	
TRPV1	0	0.6374	0.8185	
TRPV2	−0.06	0.406	0.7694	
TRPV4	0.11	0.1583	0.767	
TRPV5	0	0.5563	0.8185	
TRPV6	−0.02	0.6017	0.8185	
TRPM1	0	0.4735	0.7892	
TRPM2	0.18	0.0704	0.5817	
TRPM3	0	0.9481	0.9658	
TRPM4	−0.06	0.5091	0.8099	
TRPM5	0.1	0.1219	0.7111	
TRPM6	0.12	0.0831	0.5817	
TRPM7	−0.01	0.8858	0.9658	
TRPM8	−0.01	0.2163	0.767	

* $p < 0.05$.

Table S9. Comparison of gene expression levels between stage IV and stages I+II+III in GSE39582 dataset.

GSE39582				
**Gene**	**FC (IV vs. I–III)**	***p*-Value**	**BH-Adjusted *p*-Value**	**Significance**
KCNN1	0.02	0.467326	0.61	
KCNN2	0.06	0.534022	0.63	
KCNN3	−0.06	0.5057	0.63	
KCNN4	−0.11	0.412524	0.61	
KCNMA1	0.46	0.022382	0.19	
KCNMB1	0.39	0.053944	0.19	
KCNMB2	−0.04	0.134723	0.31	
KCNMB3	0.07	0.120564	0.31	
KCNMB4	0.14	0.329467	0.59	
ORAI1	−0.16	0.005971	0.068	
ORAI2	0.07	0.23758	0.49	
ORAI3	0.07	0.243696	0.49	
STIM1	−0.1	0.046221	0.19	
STIM2	−0.06	0.364601	0.61	
TRPA1	−0.12	0.431549	0.61	
TRPC1	0.24	0.034582	0.19	
TRPC2	0.05	0.068737	0.19	
TRPC3	0.02	0.380128	0.61	
TRPC4	−0.02	0.597806	0.68	
TRPC5	−0.01	0.753746	0.83	
TRPC6	0.03	0.468266	0.61	
TRPC7	0	0.877605	0.93	
TRPV1	NA	NA	NA	
TRPV2	−0.07	0.307387	0.58	
TRPV4	−0.07	0.12653	0.31	
TRPV5	−0.08	0.039865	0.19	
TRPV6	−0.15	0.001099	0.019	*
TRPM1	−0.02	0.399103	0.61	
TRPM2	−0.14	0.0587	0.19	
TRPM3	0	0.998509	1	
TRPM4	−0.41	<0.001	0.019	*
TRPM5	−0.09	0.065044	0.19	
TRPM6	0	0.991288	1	
TRPM7	−0.05	0.515602	0.63	
TRPM8	−0.08	0.048328	0.19	

* $p < 0.05$.

Table S10. Univariate analysis of OS and EFS in TCGA dataset.

Variable	TCGA (OS *n* = 602)			TCGA (EFS *n* = 602)		
	**Hazard Ratio**	**95% CI**	**Wald's *p*-Value**	**Hazard Ratio**	**95% CI**	**Wald's *p*-Value**
KCNN1 (High vs. Low)	0.95	(0.67–1.34)	0.755	1.09	(0.82–1.45)	0.55
KCNN2 (High vs. Low)	1.19	(0.84–1.68)	0.34	1.2	(0.9–1.6)	0.209
KCNN3 (High vs. Low)	0.92	(0.65–1.31)	0.651	0.87	(0.65–1.16)	0.33
KCNN4 (High vs. Low)	0.83	(0.58–1.17)	0.289	0.97	(0.73–1.28)	0.81
KCNMA1 (High vs. Low)	1.39	(0.98–1.98)	0.064	1.22	(0.92–1.63)	0.165
KCNMB1 (High vs. Low)	1.55	(1.09–2.21)	0.016	1.43	(1.07–1.9)	0.015
KCNMB2 (High vs. Low)	0.75	(0.53–1.06)	0.1	0.96	(0.72–1.27)	0.753
KCNMB3 (High vs. Low)	1.43	(1–2.05)	0.051	1.33	(1–1.77)	0.054
KCNMB4 (High vs. Low)	1.04	(0.74–1.48)	0.806	1.06	(0.8–1.41)	0.698
ORAI1 (High vs. Low)	1.46	(1.02–2.07)	0.036	1.24	(0.93–1.65)	0.137
ORAI2 (High vs. Low)	1.04	(0.73–1.47)	0.833	1.09	(0.82–1.44)	0.569
ORAI3 (High vs. Low)	1.72	(1.21–2.45)	0.003	1.6	(1.2–2.13)	0.001
STIM1 (High vs. Low)	1.05	(0.74–1.48)	0.8	1.11	(0.84–1.47)	0.471
STIM2 (High vs. Low)	0.7	(0.49–1)	0.05	0.69	(0.52–0.92)	0.012
TRPA1 (High vs. Low)	0.7	(0.49–1.01)	0.056	0.76	(0.57–1.01)	0.06
TRPC1 (High vs. Low)	1.44	(1.01–2.06)	0.042	1.39	(1.04–1.85)	0.025
TRPC2 (High vs. Low)	1.28	(0.9–1.82)	0.163	1.12	(0.85–1.49)	0.422
TRPC3 (High vs. Low)	0.96	(0.67–1.36)	0.807	0.96	(0.72–1.28)	0.786
TRPC4 (High vs. Low)	1.3	(0.91–1.84)	0.146	1.36	(1.02–1.81)	0.035
TRPC5 (High vs. Low)	0.79	(0.53–1.18)	0.256	1.07	(0.78–1.46)	0.674
TRPC6 (High vs. Low)	1.03	(0.72–1.46)	0.878	1.01	(0.76–1.35)	0.927
TRPC7 (High vs. Low)	1.01	(0.71–1.43)	0.962	0.98	(0.74–1.31)	0.912
TRPV1 (High vs. Low)	1.37	(0.96–1.95)	0.079	1.23	(0.93–1.64)	0.147
TRPV2 (High vs. Low)	1.06	(0.75–1.51)	0.725	1.03	(0.78–1.37)	0.83
TRPV4 (High vs. Low)	1.44	(1.01–2.04)	0.044	1.39	(1.05–1.86)	0.023
TRPV5 (High vs. Low)	1.21	(0.85–1.72)	0.281	1.2	(0.9–1.6)	0.204
TRPV6 (High vs. Low)	0.87	(0.61–1.23)	0.426	0.92	(0.69–1.23)	0.582
TRPM1 (High vs. Low)	1.26	(0.89–1.78)	0.198	1.13	(0.85–1.5)	0.416
TRPM2 (High vs. Low)	1.08	(0.76–1.53)	0.654	0.88	(0.66–1.17)	0.392
TRPM3 (High vs. Low)	1.07	(0.75–1.51)	0.717	0.95	(0.71–1.26)	0.716
TRPM4 (High vs. Low)	0.74	(0.52–1.05)	0.097	0.81	(0.61–1.07)	0.141
TRPM5 (High vs. Low)	1.45	(1.02–2.07)	0.039	1.2	(0.9–1.59)	0.217
TRPM6 (High vs. Low)	0.94	(0.66–1.33)	0.73	1.08	(0.81–1.44)	0.596
TRPM7 (High vs. Low)	0.89	(0.63–1.26)	0.503	0.93	(0.7–1.24)	0.619
TRPM8 (High vs. Low)	1.12	(0.79–1.59)	0.508	1.11	(0.83–1.47)	0.484

Table S11. Univariate analysis of OS and RFS in GSE39582 dataset.

Variable	GSE39582 (OS n = 562)			GSE39582 (RFS n = 557)		
	Hazard Ratio	95% CI	Wald's p-Value	Hazard Ratio	95% CI	Wald's p-Value
KCNN1 (High vs. Low)	1.15	(0.86–1.53)	0.336	1.16	(0.86–1.56)	0.33
KCNN2 (High vs. Low)	1.02	(0.77–1.35)	0.903	0.96	(0.72–1.29)	0.798
KCNN3 (High vs. Low)	0.95	(0.72–1.27)	0.737	0.97	(0.72–1.31)	0.858
KCNN4 (High vs. Low)	0.99	(0.74–1.31)	0.943	1.22	(0.91–1.64)	0.188
KCNMA1 (High vs. Low)	1.14	(0.86–1.51)	0.365	1.14	(0.85–1.54)	0.374
KCNMB1 (High vs. Low)	1.2	(0.9–1.6)	0.205	1.15	(0.86–1.55)	0.34
KCNMB2 (High vs. Low)	1.14	(0.86–1.51)	0.368	1.14	(0.84–1.52)	0.401
KCNMB3 (High vs. Low)	1.14	(0.86–1.52)	0.362	1.12	(0.83–1.5)	0.453
KCNMB4 (High vs. Low)	0.93	(0.7–1.23)	0.606	1.07	(0.8–1.44)	0.639
ORAI1 (High vs. Low)	0.95	(0.71–1.26)	0.697	0.86	(0.64–1.15)	0.301
ORAI2 (High vs. Low)	1.02	(0.77–1.36)	0.872	1.23	(0.91–1.65)	0.173
ORAI3 (High vs. Low)	1.12	(0.84–1.48)	0.443	1.43	(1.06–1.92)	0.02
STIM1 (High vs. Low)	0.94	(0.71–1.25)	0.685	1.05	(0.78–1.42)	0.727
STIM2 (High vs. Low)	0.91	(0.68–1.21)	0.516	0.88	(0.66–1.19)	0.405
TRPA1 (High vs. Low)	0.89	(0.67–1.18)	0.406	0.99	(0.74–1.33)	0.95
TRPC1 (High vs. Low)	1.18	(0.89–1.57)	0.258	1.34	(1–1.81)	0.05
TRPC2 (High vs. Low)	1.19	(0.89–1.58)	0.241	1.07	(0.8–1.44)	0.653
TRPC3 (High vs. Low)	1.07	(0.81–1.42)	0.63	0.99	(0.73–1.32)	0.922
TRPC4 (High vs. Low)	1.12	(0.84–1.48)	0.454	1.21	(0.9–1.63)	0.206
TRPC5 (High vs. Low)	1.05	(0.79–1.39)	0.741	1.09	(0.81–1.46)	0.576
TRPC6 (High vs. Low)	1.29	(0.97–1.71)	0.082	1.15	(0.85–1.54)	0.364
TRPC7 (High vs. Low)	1	(0.75–1.33)	0.984	1.31	(0.97–1.76)	0.077
TRPV1 (High vs. Low)	NA	NA	NA	NA	NA	NA
TRPV2 (High vs. Low)	0.96	(0.72–1.27)	0.76	1.09	(0.81–1.46)	0.575
TRPV4 (High vs. Low)	0.89	(0.67–1.18)	0.426	0.91	(0.68–1.22)	0.514
TRPV5 (High vs. Low)	0.87	(0.65–1.15)	0.324	0.93	(0.7–1.25)	0.652
TRPV6 (High vs. Low)	0.85	(0.64–1.13)	0.265	0.86	(0.64–1.16)	0.33
TRPM1 (High vs. Low)	1	(0.75–1.33)	0.998	0.85	(0.63–1.14)	0.28
TRPM2 (High vs. Low)	0.87	(0.65–1.16)	0.339	0.85	(0.63–1.14)	0.279
TRPM3 (High vs. Low)	0.94	(0.71–1.25)	0.662	1.2	(0.89–1.61)	0.227
TRPM4 (High vs. Low)	0.77	(0.58–1.03)	0.074	0.81	(0.6–1.09)	0.164
TRPM5 (High vs. Low)	0.94	(0.7–1.24)	0.651	0.85	(0.63–1.14)	0.283
TRPM6 (High vs. Low)	0.92	(0.69–1.22)	0.559	1.22	(0.9–1.64)	0.196
TRPM7 (High vs. Low)	1.17	(0.88–1.56)	0.272	1.04	(0.77–1.39)	0.811
TRPM8 (High vs. Low)	0.97	(0.73–1.29)	0.849	1.01	(0.75–1.35)	0.97

© 2019 by the authors. Licensee MDPI, Basel, Switzerland. This article is an open access article distributed under the terms and conditions of the Creative Commons Attribution (CC BY) license (http://creativecommons.org/licenses/by/4.0/).
